# Severe fever with thrombocytopenia syndrome virus replicates in brain tissues and damages neurons in newborn mice

**DOI:** 10.1186/s12866-022-02609-8

**Published:** 2022-08-20

**Authors:** Rui Chen, Qiang Li, Hongmei Chen, Hongguang Yang, Xuemin Wei, Mengting Chen, Hongling Wen

**Affiliations:** 1 School of Public Health, Wuhan University, Wuhan, China; 2 School of Health and Nursing, Wuchang University of Technology, Wuhan, China; 3 Department of Microbiological Laboratory Technology, School of Public Health, Cheeloo College of Medicine, Shandong University, Key Laboratory for the Prevention and Control of Infectious Diseases (Key Laboratory of China’s “13Th Five-Year”, Shandong University), Shandong Province Jinan, China

**Keywords:** SFTSV replication, Brain, Neuron

## Abstract

Severe fever with thrombocytopenia syndrome (SFTS) virus (SFTSV) is an emerging tick-borne phlebovirus with a high fatality rate of 12–30%, which has an expanding endemic and caused thousands of infections every year. Central nervous system (CNS) manifestations are an important risk factor of SFTS outcome death. Further understanding of the process of how SFTSV invades the brain is critical for developing effective anti-SFTS encephalitis therapeutics. We obeserved changes of viral load in the brain at different time points after intraperitoneal infection of SFTSV in newborn C57/BL6 mice. The virus invaded the brain at 3 h post-infection (hpi). Notably, the viral load increased exponentially after 24 hpi. In addition, it was found that in addition to macrophages, SFTSV infected neurons and replicated in the brain. These findings provide insights into the CNS manifestations of severe SFTS, which may lead to drug development and encephalitis therapeutics.

## Introduction

Ticks are the second largest transmission vector of pathogens after mosquitoes [1]. Infectious diseases transmitted by ticks are a growing threat to global public health[2]. Severe fever with thrombocytopenia syndrome (SFTS) is an emerging tick-borne infectious disease caused by SFTS virus (SFTSV) with the mortality rate of 6% to 30% [3]. SFTS was first recorded in China in 2009, followed by reports in South Korea, Japan, and Vietnam [3–6]. At present, SFTS cases are gradually increasing in these countries. As of 2018, a total of 11,995 cases had been reported in China alone [7]. *Haemaphysalis longhorned* tick is the main transmission vector of SFTSV, which is widely distributed in Asia, Oceania and North America [8, 9]. Epidemiological investigations showed that close contact with blood/bloody secretions of SFTS patients can also cause human-to-human transmission [10, 11]. In addition, SFTSV RNA was detected in sputum and semen, suggesting that droplets and sexual transmission may be potential transmission routes [12, 13]. Our previous study found that SFTSV could be transmitted vertically through the placental barrier in C57/BL6 mice [14]. All these findings suggest that there may be multiple transmission routes for the human-to-human transmission of SFTSV.

SFTS patients show a variety of clinical manifestations including fever, gastrointestinal manifestations (e.g., abdominal pain, vomiting, and nausea), central nervous syndromes (CNS) manifestations (e.g., dizziness, headache, encephalitis, and coma), diarrhoea, and haemorrhagic signs, among which CNS manifestations (adjusted odds ratio [OR] 30·26) are important risk factors for the fatal outcome of SFTS patients [15, 16]. The blood-cerebrospinal fluid (CSF) barrier is an important barrier to protect the CNS from pathogens, and formed by a single layer of epithelial cells of the choroid plexus [17]. CSF provides nutrients, hormones, and signaling molecules to the brain, which is necessary for maintaining the normal function of the brain [18]. However, many clinical studies detected SFTSV RNA in patients’ CSF samples and even successfully isolated SFTSV [19–21], which supported that the presence of virus in the CNS. All these findings suggest that SFTSV has the ability to damage the CNS, but there is limited research focus on the mechanism of SFTSV damage to the brain. In this study, dynamic changes of the viral loads were observed in the brain and the target cells after SFTSV infection, which provides an advanced grasp of the CNS manifestations caused by SFTSV.

## Materials and methods

### Viruses and cells

The SFTSV strain JS2011-013–1(GenBank: KC505126 to KC505128) was used in this study. TCID_50_ was determined using methods described previously [14].

### Mouse infection experiments

C57BL/6 pregnant mice were purchased from the experimental Animal Center of China Three Gorges University. Pregnant mice were raised under suitable conditions to normal delivery. One-day-old newborn mice were divided into 9 groups (*n* = 6 to 8). The newborn mice were injected intraperitoneally with the same amount of SFTSV (6 × 10^3^TCID_50_) and six mock-infected mice were used as controls. At each time point of 1 h, 3 h, 6 h, 12 h, 24 h, 2 days, 5 days, and 7 days after infection, the mice were sacrificed, their skulls were opened, and brain tissues were taken out and frozen at -80℃. The viral load and infectious SFTSV in brain tissues were tested by qRT-PCR and IFA.

### Viral load determination by quantitative real-time PCR

Frozen brain tissues were homogenized with zirconia beads in TissueLyser II (Qiagen). The viral RNA in brain tissue was extracted using Total RNA Kit I (OMEGA, Guangzhou, China) and cDNAs were generated by RT First Strand cDNA Synthesis Kit (Servicebio, Wuhan, China). The viral load was determined by quantitative Real-time PCR (qRT-PCR) with an M segment-based SFTSV-specific primer set (SFTSV-MF: AAGAAGTGGCTGTTCATCATTATTG and SFTSV-MR: GCCTTAA GGACATTGGTGAGTA). The viral load was calculated as a multiple value and expressed as log2.

### Detection of infectious SFTSV in brain tissues

Frozen brain tissues were homogenized in 150 μl DMEM, centrifuged at 15000 g for 10 min at 4℃, and 100 μl of supernatant was taken as SFTSV stock. The SFTSV stock was incubated with a monolayer of Vero cells for 2 h in a 96-well plate, and then replaced with the DMEM medium containing 0.5% FBS (Gibco) for 72 h. The cells were fixed with 4% paraformaldehyde and washed three times with PBS for 5 min each. Then, the monolayers were incubated for 1 h with human anti-SFTSV nucleoprotein (NP) mAb. After three 5-min washes with PBS, the monolayers were stained with FITC-conjugated goat anti- human IgG (H–L).

### Tissue immunofluorescence

The brain tissues of newborn mice sacrificed 7 days post SFTSV infection were submerged for 24 h in 4% paraformaldehyde at 4 °C. Tissues were paraffin embedded, and 4 μm-thick tissues sections were processed for immunofluorescence. After being blocked with 10% BSA in the assay buffer, the sections were sequentially incubated with the primary and secondary antibodies. SFTSV nucleoprotein (NP) human mAb and FITC-conjugated goat anti-human IgG (H–L) (proteintech, Chicago, USA) were used to probe SFTSV. Neun Rabbit mAb(CST, Boston, USA) and Cy3 conjugated Donkey Anti-Rabbit IgG (H + L) were used to probe neurons. F4/80 Rabbit mAb(CST, Boston, USA) and Cy3 conjugated Donkey Anti-Rabbit IgG (H + L) were used to probe macrophages. The nuclei were stained with DAPI (blue).

## Results

**Fig. 1:**
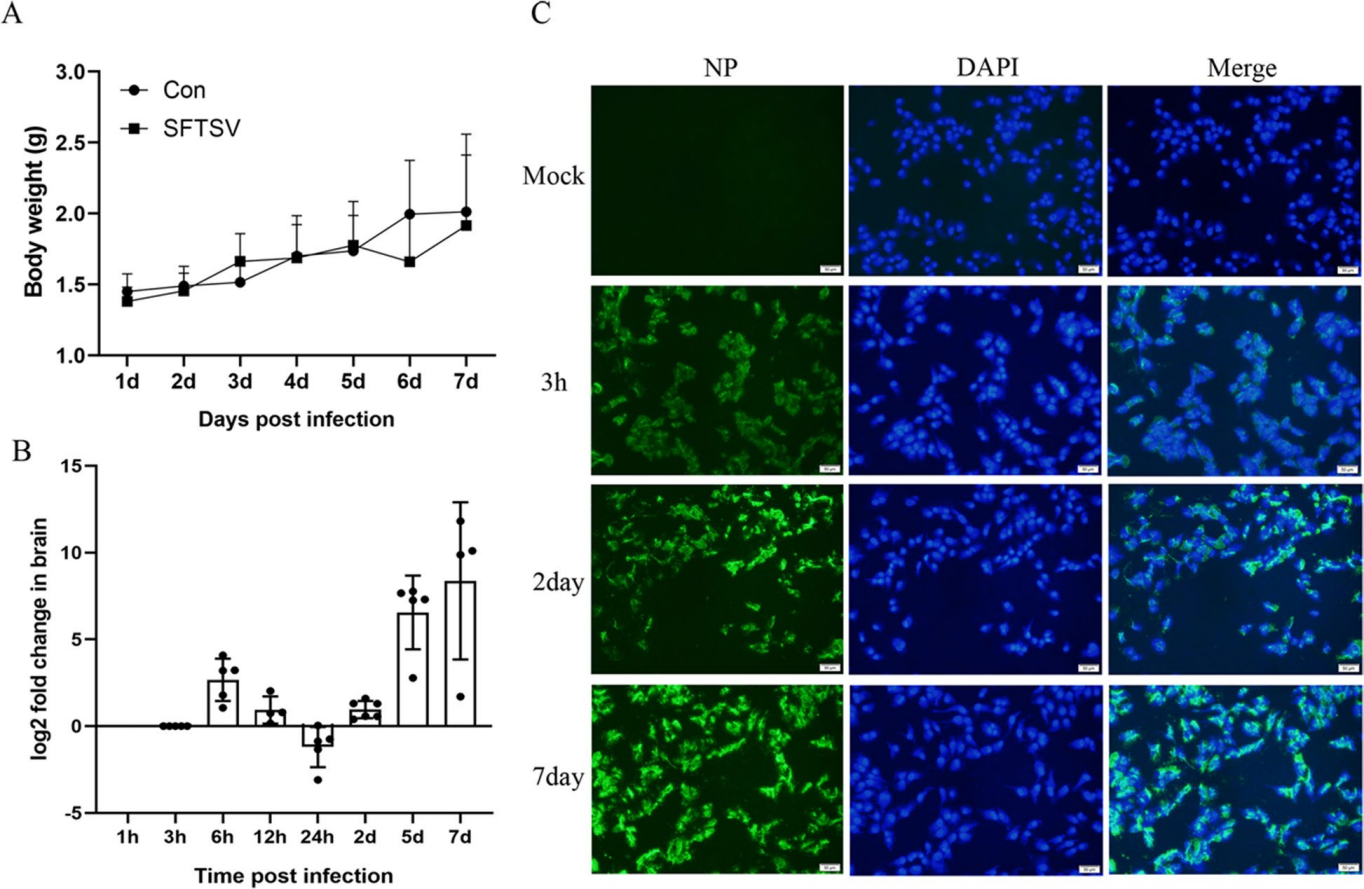
SFTSV replicates in brain tissue. **A** Weight changes of newborn mice after infection with SFTSV (*n* = 4 to 6). **B** Changes of viral loads in brain tissues of newborn mice infected with SFTSV (*n* = 4 to 6). qRT-PCR was used to detect the viral loads in brain tissues of newborn mice, and the SFTSV nucleic acid was detected at 3 hpi. The viral loads of the 3 h group were set as the benchmark, while the log2 fold changes of the other groups and the 3 h group were calculated. **C** Indirect immunofluorescence (IFA) detected the infectious virus particles in the brain tissues. Newborn mice were sacrificed at 3 hpi, 2 day post-infection (dpi), and 7 dpi, respectively. The brain tissues were homogenized, and the infectious SFTSV particles were detected by IFA. SFTSV was probed by SFTSV nucleoprotein (NP) human mAb and a FITC-conjugated goat anti-human IgG (H + L) secondary antibody (green); nuclei were stained with DAPI (blue). Bar = 50 μm

In this study, there was no significant statistical difference in the weight changes of the newbron mice infected with SFTSV (Fig. 1A). In addition, it was observed that some newborn mice died before the time of sacrifice, among which 1, 1, 1, and 2 in the 24 h, 2 days, 5 day, and 7 days groups died before sacrifice, respectively. Considering that the brain tissues of newborn mice that died prematurely were eaten by the mother mice and were not preserved in time, the viral loads were not detected. The qRT-PCR results showed that the viral RNA was detected in the brain tissues of the newbron mice after intraperitoneal injection of SFTSV 3 h (Fig. 1B). In addition, by detecting the viral loads at different time points after SFTSV infection, we found that the viral load of SFTSV reached the peak at 6hpi, decreased from 6 to 24 hpi. Notably, viral load of SFTSV increased exponentially from 24 hpi (Fig. 1B). The increase rate of viral load index in brain tissues of mice at 24 hpi after SFTSV infection suggested that SFTSV may replicate and proliferate in brain tissues. Indirect immunofluorescence (IFA) showed that there were infectious SFTSV particles in the brain of SFTSV infected newbron mice (Fig. 1C), which further confirmed that SFTSV could break through the blood–brain barrier and enter the brain tissue.

**Fig. 2:**
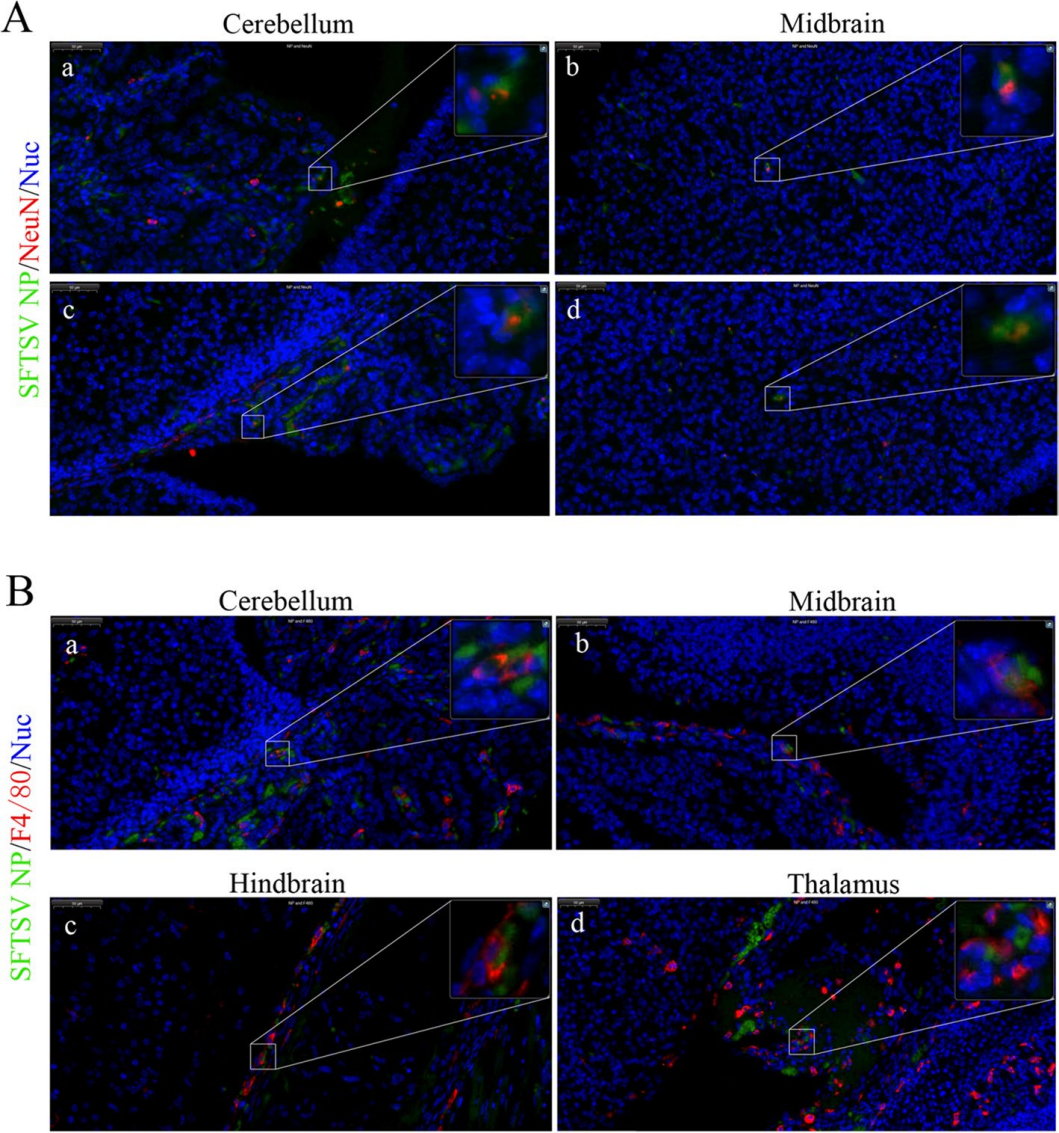
The target cells of SFTSV in the brain tissues of newborn mice. **A** colocalization of SFTSV and neurons in the brain tissues of newborn mice. a and c indicated that SFTSV infected neurons in the cerebellum; b and d indicated that SFTSV infected neurons in the midbrain. NeuN, a marker protein of mature neurons, was labeled in red. **B** Colocalization of SFTSV, and macrophage in brain tissues of newborn mice. a-d respectively indicated that SFTSV infected macrophages in the cerebellum, midbrain, hindbrain, and thalamus. The marker protein F4/80 of macrophages was marked in red. Nuclei were stained with DAPI (blue); SFTSV NPs were marked Green. Bar = 50 μm

In order to further determine the cell types of SFTSV infected brain tissues, we performed immunofluorescence analysis on the mouse brain tissues at 7 dpi. The results showed that SFTSV infected neurons in cerebellum and midbrain, as well as macrophages in cerebellum, midbrain, hindbrain, and thalamus (Fig. 2A and 2B). The local enlarged view further confirmed that neurons and macrophages were the target cells of SFTSV invaded brain tissues. These findings indicated that SFTSV infected macrophages and neurons and replicated in the brain.

## Discussion

Previous studies suggest that adult mice did not show clinical features no matter which routes adult mice were infected with SFTSV, but some newborn mice will die after infection with high-dose SFTSV [22]. Therefore, in this study, we selected the more susceptible newborn mice as the experimental animals.

The CNS manifestations caused by viral infection have always been a major public health issue of concern around the world because of more serious clinical manifestations or even death [23]. It is known that a variety of viruses can invade the brain and cause encephalitis, including zika virus, Japanese encephalitis virus, tick-borne encephalitis virus, and SARS-CoV-2, etc. [24–27]. A retrospective study of 214 COVID-19 patients in China showed that about 36.4% of patients showed nervous system symptoms, and a higher proportion of severe infection patients had CNS symptoms [28].

Since the discovery of SFTSV, researchers have drawn great attention to the CNS manifestations of SFTS patients and found evidence of imaging and laboratory tests, suggesting that SFTSV is capable to infect the CNS [19, 29]. Ning et al. conducted a clinical investigation of 538 SFTS patients and found that 19.1% of them were diagnosed with encephalitis. Notably, the SFTSV strain (HNXY-319) was successfully isolated from the CFS of one of the SFTS patients who developed encephalitis, providing direct evidence that SFTSV is capable of infecting the CNS [20]. However, due to the difficulty of collecting CNS-related clinical samples, the dynamic changes of viral loads and target cell types after SFTSV infection in the brain are currently unclear.

We found there were dynamic changes of the viral loads of SFTSV after the brain tissues were infected with SFTSV. SFTSV broke through the blood–brain barrier (BBB) of newborn mice within 3 hpi. Considering the species differences between human and newborn mice, assuming that adults have fully functional BBB, it may take longer for SFTSV to break through the adult BBB [30]. After 24 hpi, the viral load increased exponentially in the brain, which may be closely related to the differentiation of macrophages into the M2 phenotype. Previous studies found that SFTSV infected and replicated in macrophages in vivo and in vitro and aimed at virus shedding and spread by driving the differentiation of macrophages into the M2 phenotype [31, 32]. Our study revealed that in addition to macrophages, SFTSV also infected neurons in the brain. Then, considering the complexity and three-dimensional structure of the brain [33], there may be other cell types infected with SFTSV.

To our knowledge, multiple animal models have been reported to support SFTSV infection with limited symptoms of SFTS patients [22, 34–37]. For example, interferon-knockout and rapamycin-treated mice were highly susceptible to SFTSV and died within 3–4 days [36, 37]; aged ferrets infected with SFTSV mimiced most of the symptoms of SFTS patients and rapidly dies within 6–8 days [35]. The short survival time limits the application of these models to the study of pathogenic mechanisms and drug development. Furthermore, a humanized mouse model was constructed by engrafting NCG mice with human peripheral blood mononuclear cells (PBMCs) [34]. This model simulates the main symptoms of SFTS infection and exhibits longer survival time, and SFTSV RNA is detected in the brain. Newborn mice not only could be used as animal models for encephalitis research as the humanized mouse model, but also have the advantage of being more accessible.

Our findings provide pathological evidence for the appearance of CNS manifestations in SFTS patient, and emphasize the urgency of exploring the molecular mechanisms underlying CNS manifestations.

## Data Availability

The datasets used and/or analysed during the current study are available from the corresponding author on reasonable request.
